# Unlocking community capabilities for improving maternal and newborn health: participatory action research to improve birth preparedness, health facility access, and newborn care in rural Uganda

**DOI:** 10.1186/s12913-016-1864-x

**Published:** 2016-11-15

**Authors:** Elizabeth Ekirapa-Kiracho, Gertrude Namazzi, Moses Tetui, Aloysius Mutebi, Peter Waiswa, Htet Oo, David H. Peters, Asha S. George

**Affiliations:** 1 Department of Health Policy Planning and Management, Makerere University School of Public Health, Mulago Hill Road, Kampala, Uganda; 2 Department of Public Health and Clinical Medicine, Epidemiology and Global Health Unit, Umeå University, 901 87 Umeå, Sweden; 3 Division of Global Health (IHCAR), Department of Public Health Sciences, Karolinska Institute, Stockholm, Sweden; 4 Department of International Health, Johns Hopkins Bloomberg School of Public Health, Baltimore, USA; 5 South African Research Chair, School of Public Health, University of the Western Cape, Cape Town, South Africa

## Abstract

**Background:**

Community capacities and resources must be harnessed to complement supply side initiatives addressing high maternal and neonatal mortality rates in Uganda. This paper reflects on gains, challenges and lessons learnt from working with communities to improve maternal and newborn health in rural Uganda.

**Methods:**

A participatory action research project was supported from 2012 to 2015 in three eastern districts. This project involved working with households, saving groups, sub county and district leaders, transporters and village health teams in diagnosing causes of maternal and neonatal mortality and morbidity, developing action plans to address these issues, taking action and learning from action in a cyclical manner. This paper draws from project experience and documentation, as well as thematic analysis of 20 interviews with community and district stakeholders and 12 focus group discussions with women who had recently delivered and men whose wives had recently delivered.

**Results:**

Women and men reported increased awareness about birth preparedness, improved newborn care practices and more male involvement in maternal and newborn health. However, additional direct communication strategies were required to reach more men beyond the minority who attended community dialogues and home visits. Saving groups and other saving modalities were strengthened, with money saved used to meet transport costs, purchase other items needed for birth and other routine household needs.

However saving groups required significant support to improve income generation, management and trust among members. Linkages between savings groups and transport providers improved women’s access to health facilities at reduced cost. Although village health teams were a key resource for providing information, their efforts were constrained by low levels of education, inadequate financial compensation and transportation challenges. Ensuring that the village health teams and savings groups functioned required regular supervision, review meetings and payment for supervisors to visit.

**Conclusions:**

This participatory program, which focused on building the capacity of community stakeholders, was able to improve local awareness of maternal and newborn health practices and instigate local action to improve access to healthcare. Collaborative problem solving among diverse stakeholders, continuous support and a participatory approach that allowed flexibility were essential project characteristics that enabled overcoming of challenges faced.

## Background

In Uganda, maternal and neonatal mortality rates at 438/100,000 live births and 27/1000 live births respectively have barely changed during the past decade [1]. Although over 90 % of mothers attend one antenatal care visit, less than half complete the four recommended antenatal visits, and only 57 % deliver in health facilities [1]. Critical resources such as qualified health workers, drugs, equipment and supplies are needed to improve the delivery of quality health services. Community capacities and resources must also be harnessed to support maternal and neonatal health, which is the focus of this paper.

The Alma Ata declaration emphasised the importance of community participation in the planning, organization, operation and control of primary health care services [2]. Community participation and empowerment can improve access to health services and health service outcomes [3], however the literature also shows significant variations regarding the interventions implemented, the nature of the communities involved and inputs provided and how participation is defined [4], with implications for effectiveness [5]. When community participation is minimal and focused only on raising awareness of health issues, this may not necessarily improve access to skilled care services [6, 7].

Factors that can facilitate increased community participation include pre-existing intrinsic motivation among individuals in the community, community-level trust, strong external linkages, and supportive institutional processes such as decentralization reforms and engagement with social movements [4]. Conversely, community participation can be hindered by a lack of training, interest and information, along with weak financial sustainability and low community accountability [4]. Rassekh and Segeran [8] found that the most successful community engagement strategies were those that provide feedback through sharing results with communities; foster local adaptive learning; harness community resources and promote equity. These processes and factors when brought together strengthen community capability.

Community capability has been defined as “a set of dynamic community traits, resources, and associational patterns that can be brought to bear for community building and community health improvement” [9]. We followed a participatory action research approach [10], which emphasises community participation in the collaborative identification and resolution of community problems, as a key way of strengthening community capability, program relevance and effectiveness. While not comprehensively covering all domains of community capability [11], this paper provides insights into the gains realized, challenges faced and lessons learnt in supporting community efforts to improve maternal and newborn health.

## Methods

### Local context and key actors

Our work is situated in rural eastern Uganda, with a total estimated population of 1,045,100, consisting of Kamuli district (population 500,800), Pallisa district (362,600), and Kibuku district (181,700). Kibuku was carved out of Pallisa in 2010 and the two districts share similar economic activities, mainly crop farming and animal husbandry. Kamuli has more diverse economic activities, which include crop farming, animal husbandry, ranching, fishing, fish farming, bee keeping, quarrying and retail trading.

The participatory action research approach was facilitated by a project called MANIFEST (Maternal and Neonatal Implementation for Equitable Systems), which aimed at improving maternal and neonatal health. It was implemented from 2013 to 2015, following a 9 month design phase in 2012 and based on prior working relationships between key partners starting in 2009. The main goal of the project was to improve maternal and newborn health by increasing community awareness, action and access to maternal and neonatal health (MNH) services.

MANIFEST was implemented by district stakeholders with technical support provided by a Makerere University School of Public Health (MAKSPH) research team. District stakeholders involved actors at the community, parish, sub-county, and district levels. These stakeholders included leaders from the political system (local council leaders), administrative and technical system, community/religious system (priests and imams), and the district health system (from village level community health volunteers to district health teams) (Table 1). The MAKSPH team comprised of a multidisciplinary group of researchers and specialists including health systems experts, obstetricians, paediatricians, statisticians, sociologists and micro finance specialists.

**Table 1:** MANIFEST stakeholders from community to district levels

Actors involved	Description
Community	
Local council I chairperson	• Elected village leader • Lowest level in the administrative system
Village Health Team (VHT)	• A team of community health volunteers
Local transporters	• Drivers and vehicle owners who can provide transportation from villages to health facilities
Savings groups	• Support small scale savings and provide credit
Households	• Pregnant women and their spouses
Parish	
Local council II chairperson	• Elected village leader • Second level in the administrative system
Super VHT	• Supervises and supports the VHTs in a parish villages (approximately 5–10 villages per parish)
Sub county (Implementation committee)	
Local council III chairperson	• Third level in the administrative system • Political head of the sub county • Supervisory role at sub county level
Health assistant	• Responsible for prevention and control of diseases in the community • Overall supervisor of VHTs
Sub-county chief (Senior assistant secretary)	• Manages and coordinates the implementation of government policies, programmes, projects and laws at the sub county level • Sub county level coordinator
Community development officer (CDO)	• Responsible for facilitating and empowering communities for community development • Supports the saving groups within a sub county
Facility in charge of health center III	• Nurse or Clinical Officer • Responsible for providing leadership at the health facility to ensure health services are provided according to government regulations
Religious leader	• To provide leadership to religious group and encourage good morals • Mobilise communities to participate in health activities
District Implementation committee	
District political leadership	• Local council V chairperson Fourth and highest level in the administrative system • Resident District Commissioner. Representative of the president in the district • Supervisory and oversight role
District health team	• Comprised of nursing officers, health education specialist, facility managers, district health officers • Responsible for overseeing health services at district level
District administrative team	• Chief Administrative Officer, Chief Financial officer, District Community development officer and District Commercial Officer • Supervisory and oversight role

### The PAR intervention

PAR involves diagnosing a problem, planning action to address the problem, taking action and learning from this action in a cyclical manner. These stages are detailed in the sections that follow.

#### Diagnosing and planning action

It is important to include all stakeholders (especially the end users, i.e. households) in the design stage, to ensure that the solutions developed align with stakeholder needs and contexts. We conducted a series of consultation workshops and focus group discussions with community members to identify problems that women face when seeking maternal health services and to identify feasible solutions to these problems. These discussions were held along three main themes: quality of maternal health services, birth preparedness and transport for maternal health. The findings from these consultations were used to develop key components of the interventions implemented.

Following the design stage, implementation manuals and training materials were developed by MAKSPH and district based staff. These manuals and training materials were designed to be used by different implementing actors while conducting community dialogues, home visits and radio talk shows and while managing saving groups.

#### Taking action

**Fig. 1:**
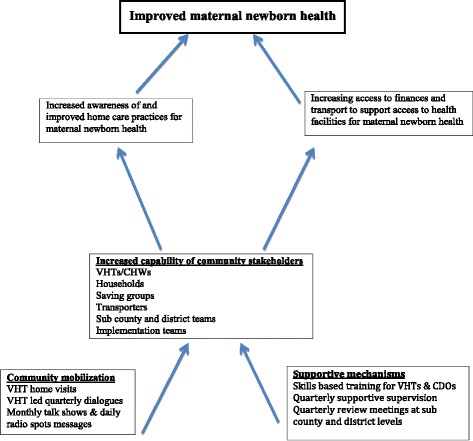
Project framework mapping community capability to improved maternal and newborn health

The project strengthened the capability of community stakeholders through community mobilization and supportive mechanisms (Fig. 1).

Community mobilization involved improving awareness about maternal and newborn health and improving maternal and newborn health practices in the home, specifically promoting birth preparedness; appropriate home care for pregnant women and newborns, and supportive male involvement. Awareness was raised through VHT home visits, community dialogues, talk shows, and radio spots. The radio sports covered a wide range of topics including: the importance of attending antenatal care, delivering in a facility, and receiving postnatal care; encouraging male involvement in maternal health; birth preparedness; the benefits of saving money to enable healthcare spending during pregnancy, childbirth and the neonatal period; and newborn danger signs, referral and caring for newborns.

The radio spots and messages, developed in conjunction with the district health educator based on feedback from the design phase, were aired on local radio stations in respective local languages. The spots were run daily and the talk shows were conducted on a monthly basis. The talk shows were delivered by the district health officials and political leaders.

Concurrently, support was provided for community development officers (public servants responsible for community development work including providing support to saving groups) and VHTs (community health workers). This support consisted of skills based training on how to assist saving groups in improving their management, their abilities to generate income and their link with local transport providers. A refresher training was conducted at the beginning of the project followed by quarterly meetings that were used to reinforce knowledge (Table 2).

**Table 2:** Types of review meetings

Type of review meetings	Frequency	Participants
1. District stakeholder meetings	Annually	DHT, political stakeholders, DCDO, implementing partners, Sub county chiefs, Opinion leaders/religious leaders
2. Sub county stakeholder meetings	Annually	Sub county implementation team (sub county chief, CDO, Health assistant), In-charge HC III, Religious/opinion leaders), Saving group representatives, local council 1 chairpersons, VHTs
3. Health work symposia	Bi-annually	DHT, district level political leaders, Health workers, health assistants.
4. District implementation team review meetings	Quarterly	DHT, DCDO, political stakeholders, Religious leader
5. Sub county review meetings	Quarterly	Sub county implementation team: politicians, technical team (sub county chief, CDO, Health assistant), In-charge HC III, Religious/opinion leaders
6. CDO meetings	Quarterly	DHT representative and CDOs
7. VHT group meetings	Quarterly	VHTs, VHT supervisors (health assistants and health workers)

In addition, quarterly supportive supervision of VHTs and quarterly review meetings at both sub county and district levels were supported to both build capacity and strengthen local accountability. As shown in Table 2, during these meetings government actors (VHTs, community development officers, health assistants, sub county and district managers) were asked by local stakeholders (politicians, religious representatives, development partners, and local council leaders) to explain cases of poor service delivery in their respective areas. The government actors were informed about these cases through their community activities such as home visits and community dialogues. Solutions to these problems were then discussed and agreed upon. The MAKSPH research team and the district teams documented these meetings, noting issues discussed, achievements and challenges.

#### Learning from action

General findings and learning events from the project were identified and documented on an ongoing basis. This included how planned activities were carried out, challenges experienced, how these challenges were resolved, and whether the objectives of the meetings or activities were met. The implementation of the program was undertaken in a phased manner to ensure learning from the roll out of the program. There was engagement with stakeholders at all levels to share experiences and lessons learned during the action cycle.

The review meetings (Table 2) allowed all those involved in implementation as well as recipients to explore their subjective experiences about the programme, actions taken, as well as mechanisms and reasons for these actions. This continuous assessment helped to inform decisions to improve outcomes of the programme and to increase chances for sustaining the initiatives. Stakeholders identified problems and suggested solutions; the program was then adjusted based on these suggestions. Table 3 provides a summary of key changes that were made and reasons for these changes.

**Table 3:** Key changes made to the programme and reasons for the changes

Component	Original design	Change made	Reason for the change
VHT component	Supervision only by health workers	Supervision by super VHTs as well	Promote sustainability of the component since health workers are few and busy
Dialogue meetings	Dialogue tools long and lengthy	Dialogue tools revised to make them shorter more focused	Feedback from VHT’s that tools were complicated
	Meetings conducted at village level, with a large group of participants making dialogue impossible	Meetings conducted in saving groups	To promote sustainability of the meetings after the project by using an existing group that convenes regularly and to promote dialogue
Savings and transport	Minimal involvement of CDOs	Increased involvement of CDOs to provide regular support to the saving groups through training and supervision	Feedback during review meetings showed that households were not saving and linkages with transporters were not being formed
	No facilitation planned for CDOs since the support is part of their regular work	Facilitation for CDOs to allow them to visit the groups and to provide the necessary support	Feedback from CDOs that they were not doing the expected work because they lacked facilitation
	No linkage with other income generating groups	CDOs were encouraged to work with other existing income generating groups in the district to leverage existing resources	Feedback about the groups accumulating a lot of savings which was now redundant and likely to be stolen
			Fears about money being stolen
			Low incomes given as a reason for not saving

### Data collection

Alongside the PAR intervention, MaKSPH undertook a series of research activities designed to evaluate and inform the project. All study procedures were documented, as well as any deviations or changes that were made, in addition to any intended and unintended positive and negative consequences and steps taken to mitigate negative consequences.

In addition, data for this paper has been drawn from key informant interviews (KIIs) and focus group discussions (FGDs). Twenty KIIs were carried out across the three districts with members of the sub county implementation committee who were involved in the implementation of the project at the beginning of the study and at the end of it, e.g. sub county chiefs, chairpersons, religious leaders, health assistants and facility in-charges. KIIs were also conducted with community leaders who were mainly involved in community mobilisation, such as local council chairmen and VHTs. In addition, KIIs were done with members of the district health team who took the lead in overseeing implementation. Written informed consent was sought from the key informants before conducting the interviews.

Furthermore, 12 FGDs across the three districts were carried out at the beginning and end of the project. The FGDs were homogenous in composition and were with women (6 FGDs) of reproductive age who had given birth during the project’s implementation and men (6 FGDs) whose wives had delivered during the same period. Each of these FGDs consisted of eight to 12 participants. The participants for the focus group discussions were chosen purposively with the help of the local council one chairpersons who are the gatekeepers in the community. Verbal informed consent was sought from the focus group participants.

All tools were translated from English to the three local languages used in the study districts i.e. Lusoga, Lugwere and Ateso by three pairs of research assistants (RAs) who speak both English and the respective languages. All FGDs were tape recorded and the notes transcribed into English. Discussions on average lasted between 1 and 1.5 h.

The qualitative data were analysed thematically. Analysis began with a detailed reading of the transcripts. Codes were then developed to identify and tag segments of text on the research topics of interest. After applying these codes to the transcripts, the researchers examined the coded text and generated broader themes that emerged from the data. The main themes in relation to key achievements were: awareness about maternal and newborn health, multi-sectorial collaboration, male involvement, improved care seeking, financial empowerment. The main themes in relation to challenges and lessons learnt were: poverty, facilitation, technical support and flexibility in the research approach. The quality checks that were implemented included the training of research assistants, pretesting of tools, field debriefing and review of data.

## Results

In this section of the paper we present the main gains, challenges and lessons learnt while supporting community capability for maternal and newborn health. We present our findings by the community stakeholders that were the focus of the project: households, transporters, saving groups and VHTs.

### Households

The main gains reported by women and men at household level included increased awareness about birth preparedness, improved newborn care and more male involvement in maternal and newborn health.

#### Maternal and newborn health awareness and care

Mothers reported that they became more aware of what they themselves could do to prepare for birth through the sensitization from the VHTs during home visits and community dialogue meetings, as illustrated in the following quotes.

*I benefited from the VHT’s visits because women have been ignorant. You would be there pregnant but when you don’t know how you will care for the baby after birth. Maybe you would just tear any rug and wrap the baby in it after delivery. But as they taught me, I started preparing birth requirements when my pregnancy was four months. (FGD women)*

*We were at a village meeting and there was some kind of sensitization. All ladies who were expecting and those with babies of not more than a year were asked to start saving. With the money we had, we were asked to start saving a certain portion to help us meet the childbirth financial costs in addition to what husbands may provide. (FGD women)*

The women also reported learning how to care for their babies. In particular key behaviours related to newborn health and survival related to hygiene and cord care were discussed.

*I have benefited because we are now informed girls who know what to do, who can practice good hygiene in our homes. These things of coming back from the garden then you just rush to carry the baby are no longer there; you have first of all to clean your hands before carrying the baby. If am in the garden, I make sure that I go with water to wash my hands when I want to breastfeed. (FGD women)*

*…Before MANIFEST came when I could give birth, my mother- in law would immediately come and say put this and that on the baby’s cord so that it heals very fast but when this program came and they started educating us on how you can take care of the baby’s cord that you just clean it with water and a little soap, we stopped all those things like putting powder and so on. (FGD women)*

#### Male involvement

During the project, men and women were encouraged to participate during home visits, at dialogue meetings and in the saving groups. Overall, women participated more than men. Nevertheless some changes were observed among some of the men. They started to support their partners by procuring more nutritious diets, purchasing birth items, and saving for childbirth, as pointed out by women during FGDs.

*My husband is really taking care of me, he feeds me well, he keeps balancing the food I eat for instance if I had kalo (millet bread) for lunch, he will make sure he buys matooke (plantains) for the next meal. But previously if it was a season for beans you would feed on that until the season ends. If you would try to say buy some sauce, he would simply say go and boil beans or get greens from the garden. Through the health education that the VHTs give us, men these days have understood and they really give support. (FGD women)*

*…They have changed my husband and he can listen to what is being discussed in the meetings and even do it because he can now buy birth items when am pregnant. And when I start getting labor pains I go to the facility and the health workers help me because I will have bought the birth requirements and now it will not be hard for me. (FGD women)*

During the qualitative interviews, men themselves noted that they were now more aware of the roles that they needed to play in ensuring mothers and newborns are safe. Men reported that they escorted their wives for antenatal care and delivery, underwent joint HIV testing, and supported birth preparedness by giving money to save for birth items and transport costs.

*The VHTs have educated us about health; they say it is not good for a woman to deliver in the village because she can easily die. So that has made us know that it is important to escort our wives to the health facilities early enough so that they can get the required services. (FGD men)*

*…At least men are also aware that saving is not only for women it’s for both, because these days men can give their women money [and say] that you take this money to the saving group. (KII with community development officer)*

Although a positive response was noted with regard to male involvement, in a few instances money saved by women was taken by their husbands and used for non-maternal and newborn issues. In the FGDs it was also noted that a minority of the men did not support their wives because of addiction to alcohol, lack of understanding about maternal and newborn health, polygamy and misunderstandings between the men and women.

*What I can say is men who don’t help pregnant and newly delivered women don’t attend meetings so they don’t know what is taking place. (FGD women)*

*They don’t support their women because they have got addicted to alcohol; they just wake up in the morning and go to bars to drink so the money that would be saved to prepare for birth or to help the newly delivered mother is all spent in drinking. (FGD Women)*

The failure of the majority of men to attend community dialogues and home visits clearly illustrated the need for communication channels that can target men more directly and the benefit of using a mix of communication channels.

### Saving groups and local transporters

#### Saving practices

Many families relied on income generating activities like rearing chicken, keeping domestic animals and running small businesses from which they accumulated some funds for saving.

*Some of us just do garden work [small scale farming]. Others sit alongside the wards and sell silver fish. So whenever she gets a small profit she adds on her savings. Other women who cannot do that just sell off what they rear at home, that could be a chicken, and then she brings the money to save. (FGD women)*

It was noted that in the past when someone had a problem, they would have no where to borrow money from, as a result of which assets were sold off when they needed money for maternal and newborn health. By strengthening saving groups, families were able to save more money than they had previously. This money was used to meet transport costs and to purchase other items needed for birth, as well as meeting other personal needs that families encountered.

Although households were encouraged to save, some of them cited lack of access to cash and low incomes as a constant reason for not saving. In addition, most of these saving group leaders lacked the necessary knowledge and skills in management and record keeping. With time, as the savings groups were strengthened, they accumulated a lot of savings but did not know how to handle these deposits. They did not know what income generating activities to invest in and at the same time feared putting their money in the bank because they had not been exposed to the benefits of using banks to keep money. They also encountered problems related to default in payment. In some cases, this led to the collapse of groups.

These challenges were countered first by encouraging the saving groups that had large sums of money to bank it. Second, we worked with the community development officers to encourage saving groups to identify income-generating activities that were likely to be successful in their community. This was done by promoting exchange visits and sharing of experiences between saving groups and groups that had successful income generating activities. The problem of default in payment was mitigated by encouraging saving groups to develop constitutions with clear criteria for lending and steps for recovering payment.

#### Linkages with transport providers

Saving groups were supported to work closely with transporters and VHTs to identify pregnant mothers and transport them to health facilities to deliver when the time came. Some of the saving groups where able to make formal and informal agreements with transporters to provide transport services to the women in the saving groups. These agreements stipulated the transport charges from specific villages to the health facilities that serve the villages.

They also indicated that the transporter was obliged to provide transport as and when required by the client (woman). This protected the women from having to pay unfair prices it also assured them of transport whenever it was required. Furthermore the women could be transported on credit and then the saving group pays later. This was critical especially in the case of referral because mobilising sufficient cash to meet the high referral costs previously led to delays in referral, sometimes with fatal outcomes for either the mother or the newborn.

The feedback received during group meetings with the transporters and VHT’s showed that saving groups that had transporters as members where more successful in convincing the transporters to provide transport services than those that did not have transporters within the group. Some of the saving groups purchased their own motorcycles and contracted transporters to ride them, in such cases members from the group were charged a lower fee.

*I really benefited. We had transporters as part of the group. So in case you belonged to that group, transport was provided at half price by the boda bodas. (FGD women)*

*For me the issue of transportation is very good because it saves lives. These transporters are paid after they – bodas have transported our women to the health facility through their savings unlike those days when they used to deliver in banana plantations. (KII with community development officer)*

Although some saving groups were able to work with the transporters as illustrated above, many saving groups were not able to identify a transporter to work with. Women from such saving groups that had no transporters resorted to using any transporter who was available in the community and this arrangement also worked well especially for routine trips to the health facility for antenatal care or delivery (not emergency referrals).

Several reasons were proposed as explanations for the difficulties encountered in working with transporters. They included difficulty in sensitizing transporters since in some cases they did not have associations and so were difficult to reach, lack of trust between transporters and saving groups, inadequate number of transporters who did not wish to be committed to only one saving group, preference for immediate payment, and fear of signing agreements (often because of high illiteracy).

When the program was designed it was assumed that after having an orientation meeting for saving group leaders and transporters, they would link up in the community and start providing the required services. However the groups were not able to organise themselves as required. Thereafter it was decided that the CDOs would be encouraged to provide more support to the saving groups. CDOs were asked to support saving groups to develop constitutions and register at the sub county and district level. In addition, CDOs were to encourage saving groups to link up with transporters. Each CDO was to ensure that he had at least one model saving group in every parish.

When the project decided to work with the CDOs to support the saving groups, it was assumed that the CDOs would be able to provide the support as they went about their other duties. However later it emerged that although the government expects CDOs to support the saving groups in their day-to-day duties, most of them had no means of transportation to the communities and no transport allowance. Some of them had motorcycles but they did not have fuel. When they were given transport allowances by the project they were able to perform their work. This illustrates that without adequate resources community level cadres may not be able to carry out their duties satisfactorily.

### Village health teams

As mentioned earlier, the VHTs were a key resource for community mobilization and sharing information with households. The home visits were conducted twice during pregnancy and two times in the first week after delivery. The VHTs visited all the homes every three months to identify new pregnancies. During the home visits the VHTs provided health education about danger signs during pregnancy, delivery and after birth, and birth preparedness.

The community dialogues were done every quarter at village level and they were facilitated by VHTs. During the dialogues the community discussed issues that were relevant and important for guiding appropriate decision making regarding improved access to MNH. They also developed suggestions of how they could solve local problems related to seeking MNH care. Women were highly appreciative of the information and guidance provided by VHTs as noted in the following quotes.

*The first time the VHT came to my home I was four months pregnant. He asked me how old my pregnancy was and I told him it was four months and he told me that if it reaches eight months, I should have already bought all the requirements. So he advised me to start saving money. He told me to buy gloves, razorblade, baby shawl, and cotton. Everything, including a basin. (FGD women)*

*You may be pregnant and at the same time have many complications in your body like swelling of legs and hands and also feeling weak all the time. So VHTs have helped us a lot to make sure that they send us to health facilities to get treatment and become well. (FGD women)*

While being valued as key community resources, VHTs faced barriers in terms of their low levels of education. Although the selection criteria for VHTs specify that a VHT should be able to read and write, some of them were not able to do so. This decreased their ability to comprehend key concepts and to keep good records. A key lesson was recognising the need for close supervision and reinforcement of knowledge, particularly in the initial stages of the project.

Another challenge that VHTs faced was that despite VHTs being volunteers, they put in a lot of time to visit homes and cover large areas in terms of distance. VHT’s received 10,000 sh (Approximately $3 USD) only as a transportation refund for conducting dialogue meetings and attending VHT group meetings every quarter (total of $6 USD). VHTs preferred more regular payments and expressed the desire to be included on the government payroll. They also expressed the need for bicycles to be able to move easily within the communities. Broader community members also felt that VHTs should be given better transportation support:

*Now these VHTs walk on foot all day, they would ease on their mobility by providing them with transport that can motivate them to work. (FGD women)*

On several occasions VHTs requested equipment, such as umbrellas for use during rainy and hot seasons. However, the project was only able to provide a t-shirt for identification purposes and bags to carry and protect their health education materials, which they appreciated.

Although under the Government system, the Health Assistants and health workers from the catchment areas are supposed to supervise VHTs, the study revealed that this does not always happen due to challenges in facilitation and workload. The project therefore provided some monetary facilitation to enable these cadres to do their work. During the first year of the program, health workers were supported on a quarterly basis to provide directly observed supervision during home visits for every VHT and quarterly supportive supervision meetings to motivate and reinforce knowledge and skills of the VHTs and VHT trainers.

The work with the VHTs also illustrated the importance of high-level participation and engagement of trainers/supervisors in creating ownership. It was observed that the VHT trainers/supervisors who were involved right from the beginning were more knowledgeable, committed, and exhibited a greater sense of responsibility than the supervisors who were brought on board later.

## Discussion

Through a participatory action research process, district teams accompanied by a research team at Makerere University, strengthened the capacity of a diverse group of stakeholders to improve maternal and newborn health. Positive results included increased awareness about birth preparedness, improved newborn care practices and more male involvement in maternal and newborn health. While women and project stakeholders stressed the value of male engagement, more direct communication strategies were required to reach additional men beyond the minority who attended community dialogues and home visits.

Saving groups and other saving modalities were strengthened, with money saved used to meet transport costs, purchase other items needed for birth and meet other personal needs. Some households however cited lack of access to cash and low incomes as a constant reason for not saving and savings groups required significant support to improve their income generation, management skills and interpersonal trust among members. Linkages between savings groups and transport providers improved women’s access to health facilities at reduced cost.

VHT’s were a key resource for providing information, however their efforts were constrained by their low levels of education, inadequate financial compensation and transportation challenges. To ensure VHT and savings groups functioning, support mechanisms in terms of regular supervision, review meetings and payment for supervisors to visit was critical.

Our experience of working with communities on improving maternal and newborn health is comparable to studies elsewhere. Other efforts to improve community-facility linkages found that, in addition to high levels of active community participation, programs that were most effective had: contextualized newborn problems in the local customs/culture; the involvement of a broad range of key community stakeholders; and home visitation and peer counselling [7].

With regards to efforts related to savings groups, communities have been known to contribute funds through loan schemes, insurance groups or other financial social networks [12–15]. These funds are used either to meet the medical (drugs, supplies, consultation fees) or nonmedical costs (transport, food etc.) of health care and have been successful in increasing the utilization of MNH services [13, 14, 16].

Community initiatives that use CHWs have led to improved maternal and newborn care practices, birth preparedness, and a decline in maternal and neonatal mortality especially in South East Asia [17–20]. The success of these CHWs depended on several factors which included community ownership and mobilization, their selection processes, training, supportive supervision, monetary incentives, their management and their link with the health system [21–24].

However, the use of CHWs has not always led to positive effects in reduction in mortality [3, 25, 26]. Positive impacts were registered where community participation interventions were part of supply side improvement packages, male partner involvement and involvement of significant others in the households. The scaling up and sustainability of these interventions should be given considerable attention during the designing of programs. Interventions that involve community mobilization and empowerment, that have been institutionalized within local structures and that meet an identified need in the community are more likely to be sustained [16, 27].

We conclude with reflections on three cross-cutting issues that were important in our efforts to work with communities to improve maternal and newborn health in rural Uganda. These include the use of a diverse group of stakeholders, provision of support to enable these stakeholders to play their roles satisfactorily and the use of a participatory approach.

Solving maternal health problems requires networking and collaboration among diverse stakeholders at multiple levels of the health systems. In our experience, local leaders (chairpersons, sub county chiefs, council members) were the entry point into the communities and they played key roles in the mobilisation of communities for community dialogues and sensitisation of the communities about the advantages of saving as a means of birth preparedness. They also played key roles in holding providers such as the district health officers and facility managers accountable.

At the household level, the involvement of men in maternal health related issues was very crucial because they hold the economic power and are the decision makers in the family and so they influence the actions taken by their wives [3, 28–31]. Some of the factors that have stopped women from accessing health services, such as heavy workload, lack of access to cash and lack of decision making power, are a result of existing gender inequalities [31].

Interventions therefore need to be designed to be gender sensitive and to promote shared responsibilities between men and women in all these areas, so that women are empowered in all these different dimensions. This intervention provided an opportunity for women to generate income by participating in saving groups, while at the same time encouraging joint decision making by providing health education about danger signs and the importance of skilled attendance to both women and their husbands. It also stressed the importance of male involvement in maternal health, for example through sharing household chores and ensuring adequate nutrition. Some of these roles had been previously seen as the responsibility of only women [31].

Indeed there is evidence that provision of education about maternal health to men and women and increased male involvement in maternal health improves birth planning and skilled attendance [32–35]. Linkages and trust between community members through saving groups and transport providers proved essential to overcome financial and geographic barriers to accessing health facilities. Programs that integrate multiple interventions for maternal health are likely to succeed, as no one intervention on its own is likely to have effect in low resource settings [16, 36].

A significant amount of human resources were needed to execute the community mobilization and empowerment activities. The project worked with existing local human resources such as VHTs, health workers and community development officers. Nonetheless, significant capacity building was required, with continuous support for problem solving and identification of feasible solutions. It was also noted that one of the reasons why these structures failed to function is because of a lack of resources and unrealistic workloads. For example, one CDO was responsible for providing support to over 80 groups with no transport support.

Similarly health assistants and health workers were expected to support VHT’s without sufficient facilitation. In addition, to supporting transport funds, the project also promoted a system of peer support with super VHT’s supporting their colleagues.

The use of community health workers, volunteers and other community resources have been seen as a way of doing things cheaply however it is important to note that for these structures to function appropriately, significant financial and human resources are required [37].

Lastly, the participatory design of the project promoted the participation of local stakeholders. It empowered them to make decisions that they felt were appropriate [37–40]. The flexibility of the design also allowed the implementation of the program to be modified in response to feedback from local stakeholders. For example when things were not working, the team was able to identify the problem and suggest a local solution that could be used to address the problem.

This paper provides valuable lessons about the contribution of communities to improving maternal and newborn health. However the data presented relied mainly on qualitative research which although useful for providing in-depth explanations was not able to show a cause and effect relationship. Another limitation of our work is that we focused on only a few domains of community capability. Lastly the intervention comprised of a package of interventions and so it was not possible to separate the different components of the intervention.

## Conclusions

Our experience demonstrates that it is possible to support existing community stakeholders and harness community resources for improving maternal and newborn health. Significant gains were realized despite the challenges faced due to a number of cross-cutting factors: the engagement of a diverse set of stakeholders, collaborative problem solving around maternal health issues, a multi-sectorial approach, the provision of continuous support and facilitation, capacity building to ensure implementers had the skills to do what was required of them, and lastly a participatory study design that allowed flexibility and change in response to feedback from the stakeholders.

We therefore recommend that similar programs work with existing local resources that meet pre-specified criteria. In addition project plans should include resources for strengthening the capacity of local structures so as to ensure that they have the capacity required to implement their responsibilities.
